# Development of the human cancer microRNA network

**DOI:** 10.1186/1758-907X-1-6

**Published:** 2010-02-02

**Authors:** Sanghamitra Bandyopadhyay, Ramkrishna Mitra, Ujjwal Maulik, Michael Q Zhang

**Affiliations:** 1 Machine Intelligence Unit, Indian Statistical Institute, Kolkata, India; 2 Department of Computer Science and Engineering, Jadavpur University, Kolkata, India; 3 Watson School of Biological Sciences, Cold Spring Harbor Laboratory, Cold Spring Harbor, NY 11724, USA; 4 MOE Key Laboratory of Bioinformatics and Bioinformatics Division, TNLIST, Tsinghua University, Beijing 100084, China

## Abstract

**Background:**

MicroRNAs are a class of small noncoding RNAs that are abnormally expressed in different cancer cells. Molecular signature of miRNAs in different malignancies suggests that these are not only actively involved in the pathogenesis of human cancer but also have a significant role in patients survival. The differential expression patterns of specific miRNAs in a specific cancer tissue type have been reported in hundreds of research articles. However limited attempt has been made to collate this multitude of information and obtain a global perspective of miRNA dysregulation in multiple cancer types.

**Results:**

In this article a *cancer-miRNA *network is developed by mining the literature of experimentally verified cancer-miRNA relationships. This network throws up several new and interesting biological insights which were not evident in individual experiments, but become evident when studied in the global perspective. From the network a number of *cancer-miRNA *modules have been identified based on a computational approach to mine associations between cancer types and miRNAs. The modules that are generated based on these association are found to have a number of common predicted target onco/tumor suppressor genes. This suggests a combinatorial effect of the module associated miRNAs on target gene regulation in selective cancer tissues or cell lines. Moreover, neighboring miRNAs (group of miRNAs that are located within 50 kb of genomic location) of these modules show similar dysregulation patterns suggesting common regulatory pathway. Besides this, neighboring miRNAs may also show a similar dysregulation patterns (differentially coexpressed) in the cancer tissues. In this study, we found that in 67% of the cancer types have at least two neighboring miRNAs showing downregulation which is statistically significant (P < 10^-7^, Randomization test). A similar result is obtained for the neighboring miRNAs showing upregulation in specific cancer type. These results elucidate the fact that the neighboring miRNAs might be differentially coexpressed in cancer tissues as that of the normal tissue types. Additionally, *cancer-miRNA *network efficiently detect hub miRNAs dysregulated in many cancer types and identify cancer specific miRNAs. Depending on the expression patterns, it is possible to identify those hubs that have strong oncogenic or tumor suppressor characteristics.

**Conclusions:**

Limited work has been done towards revealing the fact that a number of miRNAs can control commonly altered regulatory pathways. However, this becomes immediately evident by accompanying the analysis of cancer-miRNA relationships in the proposed network model. These raise many unaddressed issues in miRNA research that have never been reported previously. These observations are expected to have an intense implication in cancer and may be useful for further research.

## Background

A family of approximately 22 nucleotide (nt) noncoding RNAs termed microRNAs (miRNAs) has been identified in eukaryotic organisms ranging from nematodes to humans [1-3]. *Caenorhabditis elegans *(*C. elegans*) lin-4 and let-7 are the first discovered miRNAs [4-6]. Increasing evidence indicates that miRNAs are key regulators of various fundamental biological processes such as proliferation, apoptosis, differentiation, and so on [7]. For example let-7 family miRNAs identified in *C. elegans*, Drosophila, Zebrafish or Human [5,8,9] have important roles for terminal differentiation in normal embryonic development, temporal upregulation and so on. In let-7 mutants, stem cells can fail to exit the cell cycle and terminally differentiate at the correct time [5], so that they continue to divide which is an indication of cancer.

MiRNA genes are transcribed by RNA polymerase II (Pol II) in the nucleus to form large pri-miRNA transcripts which are capped (^7^MGpppG) and polyadenylated (AAAAA). These pri-miRNA transcripts are processed by the RNase III enzyme Drosha and its cofactor, DGCR8, to release an approximately 70-nucleotide pre-miRNA precursor product. RAN-GTP and exportin 5 transport this pre-miRNA into the cytoplasm [10,11]. Subsequently, another RNase III enzyme, Dicer, cleaves the pre-miRNA in the cytoplasm about two helical turns away from the ends of the pre-miRNA stem loop, producing a double stranded RNA. A helicase unwinds the cleaved double stranded RNA in a strand specific direction. One of the unwound strands is subsequently incorporated into a ribonuclear particle complex, RNA-induced silencing complex (RISC) [12,13] and the other one (miRNA*) is degraded. The choice of the strand relies on the local thermodynamic stability of the miRNA/miRNA* duplex. The strand whose 5' end is less stably paired is loaded into the RISC. The mature miRNA then regulate gene expression by inhibiting translation and/or by inducing degradation of target messenger RNAs [14-17]. According to the miRBase of Sanger Institute http://microRNA.sanger.ac.uk/ approximately 700 miRNAs are found in human and up to one third of the total human mRNAs are predicted to be miRNA targets. Each miRNA can target approximately 200 transcripts directly or indirectly, whereas more than one miRNA can converge on a single protein coding gene target.

Recent studies indicate that many miRNAs, referred to as onco/tumor suppressor miRNAs, are involved in the development of various human malignancies [18-21]. Aberrant expression of miRNAs contributes to carcinogenesis by promoting the expression of proto oncogenes or by inhibiting the expression of tumor suppressor genes [22-26]. For example, miR-143 and miR-145 are downregulated in colon cancer [27], miR-127 is down regulated or silenced in T24 cell (Bladder transitional carcinoma cell); on the otherhand miR-99 is overexpressed/amplified in pancreatic cancer. In the following sections an extensive review of the involvement of different miRNAs in various cancer types is first conducted and the role of miRNAs in patients survival is then elucidated.

## Involvement of miRNA in human cancer

It has been observed that about 50% of miRNA genes are localized in cancer associated genomic regions (CAGR) or fragile sites [23] as well as in minimal regions of loss of heterozygosity, minimal regions of amplification, or common breakpoint regions. Consideration of fragile sites is important as these are preferential sites of translocation, deletion, amplification, or integration of exogenous genome. MiRNAs located near fragile site could be possible targets of such genomic alterations. For example miR-15a and miR-16-1 are located at chromosome 13q14.2, a region that is known to be fragile. In fact these genes are found to be downregulated or deleted in a significant fraction of patients with B cell Chronic Lymphocytic Leukaemia (B-CLL), prostate cancer, mantle cell lymphoma and multiple myeloma [28]. It is an evidence that one or more tumor suppressor miRNAs at 13q14.2 are involved in the pathogenesis of these human cancers [29].

In various cancer malignancies approximately 200 miRNAs have been identified which are dysregulated significantly. Evidence of the involvement of such miRNAs, their implications to cancer through targeting proto oncogenes or tumor suppressor genes have been described in the following subsections.

### Lung cancer

Lung cancer is a disease in which the cells of lung tissues grow uncontrollably and form tumors. Loss or amplification of a number of miRNAs have been found related to lung cancer. A differential expression pattern of a number of miRNAs has been found in lung cancer tissues than normal lung tissues of which approximately 30% of the miRNAs are located within exons or introns of known protein coding genes [30]. In an *in vitro *experiment it has been revealed that downregulation of let-7 family in human upregulates RAS protein resulting in lung cancer [31]. In this article we have reported that a total of 64 miRNAs are differentially expressed in lung cancer among which 44 are downregulated or deleted (see Additional file 1).

### Leukemia--acute lymphoblastic, acute myeloid and chronic lymphocytic

One of the most common childhood cancer type is acute lymphoblastic leukemia (ALL) derived from the clonal proliferation of lymphoid progenitors in the bone marrow. Acute myeloid leukemia (AML) is a heterogeneous group of genetically diverse hematopoietic malignancies where frequent translocations of chromosomes are observed in general. Chronic lymphocytic leukemia (CLL) is characterized by the gradual accumulation of small mature B-cells, non proliferating cells and B-cell surface markers such as CD19, CD20 in addition to CD5 [32] in the patient. In recent years a number of miRNAs are reported to be involved in various types of leukemia. The five most highly expressed miRNAs in ALL reported in [32] are miR-128b, miR-204, miR-218, miR-331, and miR-181b-1. In CLL, the five most highly expressed miRNAs are miR-331, miR-29a, miR-195, miR-34a, and miR-29c. Among these, miR-331 is also overexpressed in ALL. Another miRNA, miR-29a is clustered with miR-29b-1. In [33], this cluster is identified as a hematopoietic-enriched cluster in human acute T-cell leukemia and human megakaryoblastic leukemia cell lines. The two miRNAs, miR-15a and miR-16-1 are located at chromosomal location 13q14.3 that is deleted in B-CLL [23]. In [34], it has been shown that miR-181a family miRNAs are expressed at relatively low levels in AML. In another experiment, it has been revealed that miR-23b is repressed and miR-221, miR-222 and miR-34a are significantly upregulated in AML specimens compared to normal bone marrow and CD34^+ ^haematopoietic progenitor cells [35].

### Breast cancer

In this article we have reported that approximately 40 miRNAs have been found to be dysregulated in breast cancer (see Additional file 1). Among these, a smaller set of miRNAs are able to correctly predict normal or tumorous breast tissue with 100% accuracy. In particular, miR-10b, miR-125b, miR-145, miR-21 and miR-155 emerged as the ones most consistently dysegulated [22]. MiR-10b, miR-125b and miR-145 may be acting as tumor suppressor miRNAs and are downregulated in breast cancer cells, while the remaining two are upregulated. This suggests that these miRNAs may be potentially acting as onco/tumor suppressor miRNAs. It is hypothesized that the miRNAs downregulated in tumor tissues might be targeting oncogenes. In fact the putative targets of miR-10b are genes such as FLT1, the v-crk homologue the growth factor BDNF and the transducing factor SHC1, and so on. These genes are known to have oncogenic functions. For miR-125b, some target genes which have potential oncogenic functions are YES, ETS1, TEL, AKT3, the growth factor receptor FGFR2 and members of the mitogen activated signal transduction pathway VTS58635, MAP3K10, MAP3K11, MAPK14, and so on. For miR-145, some of the predicted target oncogenes are MYCN, FOS, YES, FLI1, MAPK transduction proteins such as MAP3K3 and MAP4K4, and so on. It is also hypothesized that the upregulated miRNAs might be targeting tumor suppressor genes. For example potential targets of miR-155 are the tumor suppressor genes SOCS1 and APC. It was found that growth inhibition in breast cancer cell line MCF-7 is possible due to the transfection of anti-miR-21 (chemically modified oligonucleotides). Another miRNA miR-9-3 is downregulated in breast cancer with either high vascular invasion or presence of lymph node metastasis [22]. A recent experimental evidence suggests that miR-205 can be considered as a potential breast tumor suppressor gene that directly targets HER3 receptor. HER3 overexpression is a hallmark of a particularly aggressive subset of breast tumors [36].

### Pancreatic cancer

Pancreatic cancer is one of the most common cancer type with a very poor prognosis, partially due to its very low accessibility to resection and resistance to chemoradiotherapy [37]. An miRNA based treatment of pancreatic cancer thereafter appears to be an important alternative towards cancer therapy as miRNAs can be directed against pancreatic cancer through various pathways, including the inhibition of overexpressed oncogenes, suppressor of tumor growth and enhancement of apoptosis. In our present study we found that, 46 miRNAs are dysregulated in pancreatic cancer among which a significant number of miRNAs are overexpressed (*P *= 6.639 × 10^-13^, for details see Table 1) showing that miRNAs tend to be upregulated in pancreatic tumor tissue. In [38], it has been reported that miR-99, miR-100, miR-100-1, miR-125a, miR-125b-1, miR-199a-1 and miR-199a-2 are overexpressed both in pancreatic cancer and chronic pancreatitis compared with normal pancreatic tissue indicating that these can be a common inciting event for neoplastic growth.

**Table 1:** Cancer tissues where significant number of miRNAs are either up or downregulated

Cancer type	% of miRNAs	Dysregulation patterns	*P*-value()
Brain	94.04% (79/84)	Downregulated	7.106 × 10^-16^
Hematologic	100% (32/32)	Downregulated	1.642 × 10^-8^
Kidney	100% (4/4)	Upregulated	3.249 × 10^-2^
Lymphoma	100% (9/9)	Upregulated	4.228 × 10^-4^
Prostate	83.33% (15/18)	Upregulated	4.406 × 10^-4^
Pancreas	91.30% (42/46)	Upregulated	6.639 × 10^-13^
Stomach/Gastric	90.90% (10/11)	Upregulated	1.205 × 10^-3^
Thyroid	81.81% (9/11)	Upregulated	1.117 × 10^-2^

### Colon cancer

In [27], two mature miRNAs miR-143 and miR-145 are identified as reduced steady state level at the adenomatous and cancer stages of colorectal neoplasia compared to normal cell. Recently on the basis of in vivo investigation [39] it was observed that in the patients group of complete response, partial response and no change after chemotherapy the expression level of let-7g is notably lower than the disease progression group. Based on the work [39], the hypothesis is that let-7g may be a significant indicator for chemo response to 5-FU based antimetabolite S-1 based chemotherapy in colon cancer. In the present study a set of 76 miRNAs are identified that are differentially expressed in colon tumor tissues or cell lines.

### Prostate cancer

Prostate cancer is the most common form of cancer among men. Current diagnostic test for prostate cancer is the prostate specific antigen (PSA) test. PSA test, however, suffers from high false positive rate. Moreover, after PSA test is positive a painful and costly biopsy is required to confirm the presence of cancer. Recently researchers have begun analyzing the miRNA expression profiles of normal and tumor samples for the diagnosis of prostate cancer. In [21], expression profiling revealed that a number of miRNAs are differentially expressed in prostate cancer tissues including miR-21. MiR-21 is overexpressed in various types of cancer indicating that miR-21 is involved in tumor growth, invasion and metastasis. However, the role of miR-21 in prostate cancer is poorly understood. In [40], the effects of miR-21 on prostate cancer cell proliferation, apoptosis, and invasion were examined. The study suggested that miR-21 could promote apoptosis resistance, motility, and invasion in prostate cancer cells and these effects of miR-21 may be partly due to its regulation of target genes such as PDCD4, TPM1, and MARCKS.

### Uterine leiomyoma

Uterine (pertaining to the uterus) leiomyoma (ULM) is the most common neoplasms in women of reproductive age. Although causes of this disease is still not fully understood but genetic alterations have been observed. Near about 40% of ULMs contain simple non random chromosomal anomalies [41]. Some genes such as estrogen receptor α, IGF1, high mobility group genes (HMG) HMGA1 and HMGA2 and COL4 family members are implicated in ULM. It is found that these genes have multiple miRNA target sites in their 3' UTRs [42,43]. In [44] a distinct miRNA expression profile has been observed between black and white ULM patients. Mir-23a/b, let-7, miR-145, miR-197, miR-411 and miR-412 are overexpressed in black women ULM patients than white women ULM patients. The differential expression of miRNAs between large (≥ 10 cm), medium (4-9 cm) and small (≥ 3 cm) tumor sizes was examined and notably 17 miRNAs have significant differences based on size of the tumor. Five miRNAs of the let-7 family are overexpressed in ULM compared to matched myometrium. Interestingly higher expression of these miRNAs was also observed in small ULM than large ULM. It has also been observed that several miRNAs such as- miR-208, miR-381 and miR-339 exhibit significant differences by age. In the present study we have reported a set of 44 miRNAs that are dysregulated in ULM.

### Thyroid cancer

One of the most common cancer in thyroid tissue is papillary thyroid carcinoma. In papillary thyroid carcinoma, the three strongly overexpressed miRNAs miR-146, miR-221 and miR-222 have a common target gene KIT. KIT is an important tyrosine kinase receptor in cell differentiation and growth and its transcript level is known to be extremely low [45,46]. An interesting observation is that in some cases miR-221 is not only overexpressed in papillary thyroid tumor tissue but also overexpressed in normal thyroid tissue which is adjacent to papillary thyroid carcinoma, suggesting that this might be an important thyroid cancer marker.

### Liver cancer

In [47] a predominant upregulation has been shown for miR-370, miR-299 and miR-125b which putatively affects the expression of BAX/AKT, *β*-catenin, IGF R type I, respectively. BAX (Bcl2- associated X protein), is a proapoptosis gene of the Bcl-2 family and it is well known that proteins of Bcl-2 family are the key regulators of apoptosis [48-50]. Lower expression of BAX is found in esophageal squamous cell carcinoma, hepatocellular carcinoma (HCC), breast cancer and ovarian cancer [51-54]. Another liver specific miRNA, miR-122, is significantly down regulated in liver cancer with intrahepatic metastasis. ADAM17 is a gene involved in metastasis is a potential target of miR-122 [55]. Silencing of ADAM17 by miR-122 resulted in a dramatic reduction of in vitro migration, invasion, in vivo tumorigenesis, angiogenesis, and local invasion. A set of 22 miRNAs are differentially expressed in HCC reported in our present study (see Additional file 1).

### Stomach cancer

Cancer that forms in tissues lining the stomach is called stomach cancer, also called gastric cancer. Stomach cancer is a polygenic disease with poor prognosis indicated by several published literature [56]. A set of 11 miRNAs are reported in the *cancer-miRNA *network that are dysregulated in stomach tumor tissue among which 10 are significantly upregulated.

### Brain and spinal tumors

Glioblastoma multiforme is the most malignant type of astrocytoma, composed of spongioblasts, astroblasts and astrocytes. It usually occurs in the brain but may occur in the brain stem or spinal cord. MiR-21 is overexpressed in glioblastoma tissues and cells and acts as an antiapoptotic factor in cultured glioblastoma cells [57]. Twelve early passage cultures from high grade gliomas and six glioblastoma cell lines (A172, U87, U373, LN229, LN428 and LN308) were compared to non-neoplastic glial cells and a variety of mammalian tissues. From the results miR-21 was found to be strongly overexpressed in the neoplastic samples.

### AIDS associated cancer kaposi's sarcoma

AIDS associated Kaposi's Sarcoma (KS) is the most commonly reported cancer in parts of Africa and it is increasingly spreading out in other parts of the world where HIV-1 infection is increasing day by day [58]. Kaposis sarcoma-associated herpes virus (KSHV) is the causative agent of Kaposi's Sarcoma and two B cell derived cancers namely primary effusion lymphoma (PEL) and multicentric Castleman's disease [59-61]. Twelve miRNAs have been identified in KSHV genome [62]. The hypothesis is that the host/cellular genes targeted by KSHV encoded miRNAs play a role in viral pathogenesis [63]. Thrombospondin 1 (THBS1), that is known to be antiproliferative, a protein inhibitor of angiogenesis, and a strong immune stimulator that can regulate T cell migration through extracellular matrix, is a potential target of multiple KSHV miRNAs and is translationally repressed in the presence of KSHV miRNAs like miR-k12-1, miR-k12-3-3p and miR-k12-6-3p. It was predicted by miRanda to have 34 binding sites for 12 KSHV miRNAs [64]. Downregulation of THBS1 can cause human cancer and may aid in immune evasion of KSHV infected cells. S100A2 and PRG1 are two other target genes that are downregulated in response to miRNAs and have link with cancer. A recent study [63] provided a list of KSHV miRNA targets but potential targets in all cell types such as lymphoid, epithelial and endothelial cells, infected by KSHV are yet to be discovered.

### Esophageal cancer

A study regarding miRNA expression in esophageal cancer revealed that miR-203 and miR-205 have changed expression in squamous cell carcinomas and esophageal adenocarcinomas compared with normal squamous epithelium. MiR-203 and miR-205 are also reported as upregulated in lung cancer [30]. A predicted target of miR-203 is the gene ECRG4 that has already been implicated in esophageal cancer.

## Involvement of miRNAs in patient's survival

It has been hypothesized that miRNAs have a deep correlation with patient's survival. A report shows that the expression level of Dicer is reduced in a fraction of lung cancers and have a prognostic impact on the survival of the surgically treated patients [65]. A univariate Cox proportional hazard regression model with global permutation test in BRB-Array-Tools indicated that eight miRNAs (miR-155, miR-17-3p, miR-106a, miR-93, let-7a-2, miR-145, let-7b, and miR-21) were related to the adenocarcinoma patients survival. Patients with high expression of either miR-155, miR-17-3p, miR-106a, miR-93 or miR-21 and low expression of either let-7a-2, let-7b or miR-145 were found to have a significantly worse prognosis. In [30], the survival analysis among the 41 stage I adenocarcinoma patients revealed that three miRNAs (miR-155, miR-17-3p, and miR-20) were associated with patients survival. It has been observed in pancreatic cancer that high expression of miR-196a-2 and miR-219 can cause of a lower survival of the patients (14.3 months and 13.6 months) than the low expression (26.5 months and 23.8 months) [38]. It is also anticipated that miRNAs may act as potential diagnostic marker of specific cancer type. An interesting observation is that in some cases miR-221 is not only overexpressed in papillary thyroid tumor tissue but also overexpressed in normal thyroid tissue which is adjacent to papillary thyroid carcinoma, suggesting that this might be an important thyroid cancer marker. In another investigation miR-99, miR-100, miR-100-1/2, miR-125a, miR-125b-1, miR-199a-1 and miR-199a-2 are overexpressed both in pancreatic cancer and chronic pancreatitis compared with normal pancreatic tissue indicating that these can be a common inciting event for neoplastic growth [38].

## Cancer-miRNA network

Several miRNAs are differentially expressed in various cancers, suggesting common altered regulatory pathways. Regulatory mechanisms of miRNAs can be well understood by identifying functional modules of miRNAs and their target mRNAs. It is known that if genes are associated with the same disorder they share common cellular and functional characteristics and their protein products have been shown to participate in the same cellular pathway, molecular complex or functional module [66-68]. If genes are linked with certain disorder associations, their protein products should interact more likely with one another within such disordered modules than with other proteins [69]. In the same way we can hypothesize that if the miRNAs are associated with the same cancer type with similar dysregulation pattern, then their target genes may share common cellular and functional characteristics and the miRNAs may have common target onco/tumor suppressor genes. Therefore, in order to globally observe and identify the miRNAs and associated cancer modules, generation of a *cancer-miRNA *network is crucial.

The *cancer-miRNA *network model has been generated based on the bipartite graph theoretic approach. We formed a bipartite graph *G *= (*U, V, E*) where *U *is the set of cancer types, *V *is the set of miRNAs and (*u, v*) ∈ *E *iff *v *is differentially expressed or dysregulated in cancer type *u*. In other words a bipartite graph based network model is constructed consisting of two disjoint sets of nodes where edges only exist between nodes from different sets. Here *U *is a set of cancer types and *V *is a set of cancer associated miRNAs. We have integrated an additional information into our network model by associating 'solid' or 'dotted' edges. A 'solid' edge means the miRNA is upregulated in the associated cancer type whereas a 'dotted' edge shows it's downregulation.

**Figure 1:**
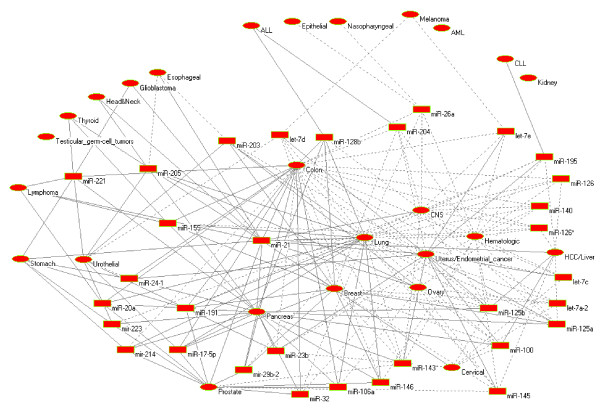
**Hubs of the network**. Solid edge = overexpression/amplification, dotted edge = underexpression/deletion.

The complete network is provided in a tabular form in (Additional file 1). Figure 1 shows a part of the *cancer-miRNA *network for illustration. As is evident, a particular type of cancer may be associated with the dysregulation of several distinct miRNAs and conversely dysregulation of one miRNA can be associated with several cancer types. The differential expression patterns of experimentally verified human miRNAs in different cancer and normal tissue types obtained from extensive literature search are taken into account. Other relevant parameters that have been considered are location of the miRNAs at fragile sites and cancer associated genomic regions, epigenetic alteration of miRNA expression and abnormalities in miRNA processing target genes and proteins.

## Novel biological insights from the cancer-miRNA network

The *Cancer-miRNA *network shown in Additional file 1 throws up several new and interesting biological insights which were not evident in individual experiments but become evident when studied in the global perspective.

### Tissue specific dysregulation patterns of miRNAs

Earlier it was believed that miRNAs are globally downregulated in tumor tissues [18]. However, current research [21] shows that this may not be true. The *cancer-miRNA *network also establishes that dysregulation patterns of miRNAs may be both up as well as downregulated. However, the *cancer-miRNA *network throws up an interesting observation that dysregulation patterns of miRNAs are tissue specific. A list of cancer types are extracted from the network where a significant number of miRNAs show either upregulation or downregulation in that specific tissue type (see Table 1). For example, in brain (central nervous system and glioblastoma) 94% of the miRNAs (79/84) are downregulated (*P *= 7.106 × 10^-16^, Fisher's exact test), and in hematogic, all the miRNAs (32/32) are downregulated (*P *= 1.642 × 10^-8^, Fisher's exact test); on the other hand 91.3% of the miRNAs (42/46) are upregulated in pancreatic cancer (*P *= 6.639 × 10^-13^, Fisher's exact test) and all the miRNAs (9/9) are upregulated in lymphoma (P = 4.228 × 10^-4^, Fisher's exact test). To illustrate this issue, we have considered individual cancer types from the network and extracted the expression patterns of the associated miRNAs from the literature, these are included in Table 1. The terms such as 'deleted' or 'underexpressed' are considered as constituting the down regulation group, and terms such as 'amplified' or 'overexpressed' are considered as constituting the upregulation group.

### Hubs and cancer specific miRNAs

In Figure 1, it can be observed that several miRNAs act as hubs, showing a significant dysregulation in several cancer types. One miRNA in the network is considered as hub if the miRNA is dysregulated in significant number of cancer types (here, five or more cancer types are considered, *P *< 10^-16^, randomization test). A hub miRNA may show homogeneous or heterogeneous expression pattern in multiple cancer types. Depending on the edges pattern of homogeneous hub miRNA, the *cancer-miRNA *network efficiently identify strong oncogenic or tumor suppressor miRNAs. For example, miR-21 is upregulated in all it's associated cancer types (except Hematologic) and shows a strong oncogenic characteristic. In this article, each cancer-miRNA link is validated by the experimental evidence. For example, the link between miR-21 and cholangio carcinoma is established based on the survey of the following information from the literature. In [70] miR arrays were performed on five primary cholangio carcinomas and five normal bile duct specimens. Differential expression of miR-21 in these 10 specimens was verified by quantitative reverse transcriptase polymerase chain reaction (qRT-PCR). To validate these findings, qRT-PCR for miR-21 was then performed on 18 additional primary cholangio carcinomas and 12 normal liver specimens. MiR-21 was 95% sensitive and 100% specific in distinguishing between cholangio carcinoma and normal tissues. As that of miR-21, miR-17-5p is overexpressed (shown with solid lines in Figure 1) in their associated tumor types, showing strong oncogenic characteristics. On the other hand let-7a-2 is downregulated in all it's associated cancer types, showing a strong tumor suppressor characteristic (shown in dotted lines in Figure 1).

In the network, a number of miRNAs are found that are associated with ≤ 2 cancer types. These miRNAs can be considered to be tissue specific. For a few miRNAs, evidence of them being tissue specific in nature is available in the literature. Though ≈100 miRNAs are identified as tissue specific miRNAs from the network, we anticipate that for several of them, this may be due to a lack of experimental evidence. As research is going on, these miRNAs may also be associated with more number of tumor tissues or cell lines and would not be recognized as tissue specific miRNAs. A full list of possible tissue specific miRNAs, its expression patterns and associated tumor types are shown in (Additional file 2).

### Identification of cancer-miRNA module using computational approach for mining association

The *Cancer-miRNA *modules are extracted from the *Cancer-miRNA *network based on a computational technique of mining associations between miRNAs and cancer types. Association rule mining (ARM) is an important tool to extract frequent patterns, associations or subgroups among sets of items from the data repository [71]. In this study, a comprehensive analysis of the combinatorial nature of miRNA dysregulation in cancer tissues has been carried out by detecting rules that identify a set of miRNAs dysregulated in several cancer types. For each rule, the scope and the precision of the rule is measured by the support and confidence statistics, respectively. To limit the extraction of statistically significant association rules, only the rules with support and confidence exceeding a predefined minimum support and minimum confidence threshold are extracted. In Table 2, the top five rules are extracted with minimum support and confidence of 0.3 and 0.8 respectively (*P *< 2.2 × 10^-16^, randomization test). However, in order to extract potential rules the threshold needs to be set pretty low since, till date, due to limited experimental validation each miRNA is found to be dysregulated only in a few cancer tissue types. As research is going on, more number of such rules are expected to be observed with greater support and confidence. In order to test the validation of our prediction, including the top five rules, we have also extracted some rules (bottom three rules in Table 2) from the network that didn't meet the predefined cutoff values mentioned above. Combining with the *cancer-miRNA *network data set the obtained rules are then observed in a set of test data set. The test data is a set of articles that were not used in anyway to build the *cancer-miRNA *network or are published after the submission of this article.

**Table 2:** MiRNAs and associated cancer types in selected cancer-miRNA modules.

Rule#	Cancer-miRNA modules		
	
	miRNAs	Cancer types (Dysregulation patterns)	Clustered miRNAs
1	{miR-100} → {miR-125b}	breast(D,D), cervical(D,D), HCC(U,U), lung(D,D), ovary(D,D), pancreas(U,U), uterus(U,U)	(miR-100, miR-125b)
2	{miR-125b, let-7a-2} → {miR-21}	breast(D,D,U), brain(D,D,U), hematologic(D,D,D), cervical(D,D,U), colon(D,D,U), lung(D,D,U), ovary(D,D,U)	(miR-125b, let-7a-2)
3	{miR-125a} → {miR-125b}	breast(U,D), brain (D,D), HCC(D,U), hematologic (D,D), lung(D,D), pancreas(U,U), uterus(U,U)	-
4	{miR-143} → {miR-21}	breast(D,U), brain (D,U), cervical(D,U), colon(D,U), hematologic(D,D), lung(D,U), pancreas(U,U), prostate(D,U), uterus(U,U)	-
5	{miR-143, miR-145} → {miR-21, miR-125b}	breast(D,D,U,D), colon(D,D,U,D), hematologic(D,D,D,D), lung(D,D,U,D), uterus(U,U,U,U)	(miR-143, miR-145)
6	{miR-17-5p, miR-20a} → {miR-143, miR-145}	colon(U,U,D,D), lung(U,U,D,D), prostate(U,U,D,D)	(miR-17-5p, miR-20a), (miR-143, miR-145)
7	{let-7a-1, let-7d, let-7f-1} → {miR-17-5p}	urothelial(D,D,D,U), colon(D,D,D,U), lung(D,D,D,U)	(let-7a-1, let-7d, let-7f-1)
8	{miR-101, miR-210} → {let-7a-2}	breast(D,U,D), lung(D,U,D), ovary (D,D,D)	-

In column 3, *D *= downregulated and *U *= upregulated for the corresponding miRNAs mentioned in column 2

In each module a number of miRNAs are found that are dysregulated in several cancer types. For example, in module two, all the miRNAs are dysregulated in hematologic, breast, brain, cervical, colon, lung and ovary. The rule of this module depicts the fact that if miR-125b and let-7a-2 are dysregulated in a cancer tissue or cell line then miR-21 is also likely to be dysregulated in that cancer tissue (confidence = 1). A number of modules in Table 2 consists of at least one miRNA cluster (group of miRNAs that are located within 50 kb of genomic location form miRNA cluster [72] or called neighboring miRNAs). Earlier works provided us clues that neighboring miRNAs may be associated with the same disease [73] such as cancer, cardiovascular disaese, Parkinson's disease, and so on. However, it is unclear whether neighboring miRNAs have differential coexpression patterns in a specific cancer tissue. In this regard, we observed the dysregulation patterns of the miRNAs in the selected cancer tissues (see Table 2). This clearly elucidates the fact that miRNAs from the same cluster have the differential coexpression patterns. For example, in module one (confidence = 1), miR-100 and miR-125b are from the same cluster having similar dysregulation patterns in breast, cervical, HCC, lung, ovary, pancreas and uterus. In test data set also, these two miRNAs are showing differential coexpression patterns (in urothelial, both are upregulated). Analogous observations are made for modules two to eight (see Tables 2 and 3). In module two, miR-125b and let-7a-2 are from the same cluster and have downregulation in seven cancer types, whereas miR-21 which is not in the same cluster shows upregulation in six out of seven cancer types. In the test set, the clustered miRNAs are showing differential coexpression pattern in HCC. In module six, two miRNA clusters (miR-17-5p and miR-20a) and (miR-143 and miR-145) are dysregulated in colon, lung and prostate cancer tissues. Notable that intra cluster miRNAs have shown similar dysregulation patterns in these cancer tissues whereas inter cluster miRNAs have the opposite expression patterns. In the test set, the intra cluster miRNAs are showing differential coexpression pattern in leukemia.

**Table 3:** Validated cancer-miRNA modules

Rule #	Cancer-miRNA modules		
	
	miRNAs	Cancer types (Dysregulation patterns)	Clustered miRNAs
1	{miR-100}→ {miR-125b}	urothelial(U,U)	[75]
2	{miR-125b, let-7a-2} → {miR-21}	HCC(D,D,U)	[76]
4	{miR-143}→ {miR-21}	oral squamous cell carcinoma(D,U)	[77]
4	{miR-143}→ {miR-21}	HCC(D,U)	[76]
5	{miR-143, miR-145} → {miR-21, miR-125b}	oral squamous cell carcinoma(D,D,U,D)	[77]
5	{miR-143, miR-145} → {miR-21, miR-125b}	prostate(D,D,U,D)	[21,78-80]
5	{miR-143, miR-145} → {miR-21, miR-125b}	HCC(D,D,U,D)	[76]
6	{miR-17-5p, miR-20a} → {miR-143, miR-145}	leukemia(U,U,D,D)	[81,82]
8	{miR-101, miR-210} → {let-7a-2}	colon(D,U,D)	[83-85]
8	{miR-101, miR-210} → {let-7a-2}	prostate(D,U,D)	[83,86]
8	{miR-101, miR-210} → {let-7a-2}	melanoma(D,U,D)	[42,83,84,87]
8	{miR-101, miR-210} → {let-7a-2}	HCC(D,U,D)	[76]

We then investigated dysregulation patterns of neighboring miRNAs in all the cancer tissues. We found that 67% of the cancer types have at least two neighboring miRNAs showing downregulation which is significantly higher (*P *< 10^-7^, Randomization test, for details see Methods) than the randomly generated data set. A similar result is obtained for the neighboring miRNAs showing upregulation in specific cancer type. We have also measured the *P *values by varying the definition of cluster (within 50 kb) and consistent result is obtained (*P *< 10^-7^, Randomization test). These results elucidate the fact that the neighboring miRNAs might be coexpressed in cancer tissues as in normal tissue types.

### Characteristics of a typical cancer-miRNA module

**Figure 2:**
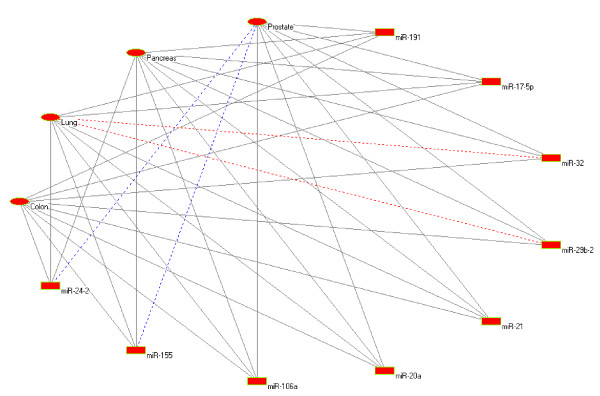
**Cancer-miRNA module**. Solid edge = overexpression/amplification, dotted edge = underexpression/deletion, blue dotted edge = unknown.

It has been observed that miR-32, miR-29b-2, miR-21, miR-20a, miR-191, miR-17-5p, miR-106a, miR-155 and miR-24-2 all have significant dysregulation in lung, colon and pancreatic cancer cell line. Thus they can be considered to form a *cancer-miRNA *module as shown in Figure 2. Among these, the first seven miRNAs also have a significant dysregulation in prostate tumor tissue but interestingly no relationship between prostate tumor and miR-155 and miR-24-2 is known. However, from the network it appears likely that these two miRNAs might be implicated in prostate tumor. This observation makes it imperative to investigate the expression pattern of miR-155 and miR-24-2 in prostate cancer cell line.

We also investigated whether the miRNAs involved in the module shown in Figure 2 have common target onco/tumor suppressor genes. As shown in Table 4 we found that most of the miRNAs in the module have some common predicted target onco/tumor suppressor genes. For example, APC is involved in colon and pancreatic cancer and it is a predicted target of miR-17-5p, miR-32, miR-20a and miR-106a. EP300 is involved in colon and pancreatic cancer (predicted target of miR-17-5p, miR-32, miR-20a and miR-106a), and so on. Beside these common target genes, we also found a number of potential target genes for each of the miRNAs that clearly shows the involvement of these miRNAs in the four cancer types, mentioned in the module. For example, TCF7L2, NRAS, VEGF and HOXC8 are the target genes of miR-191 which are involved in colon, lung, pancreatic and prostate cancer respectively. Some prostate cancer causing target genes for miR-155 such as PTGER2, MSH2 and VIM suggests the involvement of miR-155 in prostate cancer. These target onco/tumor suppressor genes of miR-155 indicates the possibility of dysregulation of miR-155 in prostate cancer cell line. This suggests a combinatorial effect of the module associated miRNAs on target gene regulation in selective cancer tissues or cell lines.

**Table 4:** Predicted common target onco/tumor suppressor genes involved in four types of cancer.

Target onco/tumor suppressor genes	Type of cancer			
	
	Colon	lung	pancreas	prostate
APC	17-5p, 32, 20a, 106a		17-5p, 32, 20a, 106a	
EP300	17-5p, 32, 20a, 106a		17-5p, 32, 20a, 106a	
DNMT1	17-5p, 32, 106a			
MSH3	17-5p, 20a, 106a			
RB1		17-5p, 20a, 106a		
HOXB4		17-5p, 20a, 106a		
RECK			21, 106a, 155	
ETV1				17-5p, 20a,106a
SYT7				17-5p, 20a,106a
EGR1				191, 32, 106a
PTEN				17-5p, 21, 20a,106a
MCL1				17-5p, 32, 20a,106a
STAT3				17-5p, 21, 20a,106a

Table should be read in this way: APC is a target gene of miR-17-5p, miR-32, miR-20a and miR-106a and this gene is involved in colon and pancreatic cancer

Another important observation from Figure 2 is that all the miRNAs except miR-155 and miR-24-2 are overexpressed in colon, pancreatic and prostate cancer. Except miR-29b-2 (which is down regulated) and miR-32 (which is deleted), these are also overexpressed in lung cancer. This might suggest further experimentation with miR-29b-2 and miR-32 to either establish or correct this observation. These information could be helpful for further research on the implication of miRNAs in cancer. As the amount of evidence of the involvement of human miRNAs in cancer grows day by day, the utility of such a *cancer-miRNA *network will be more evident in future.

## Conclusions

Recent evidence indicates that miRNAs have important roles in human malignancies and act as onco/tumor suppressor miRNAs. The cancer associated genomic regions, putative and experimentally verified target onco/tumor suppressor genes, significant over or underexpression of the miRNAs in specific cancer tissues or cell lines are a few potential evidences of the involvement of miRNA in cancers. In this article an indepth survey has been conducted on the dysregulation of specific miRNAs in different cancers. This leads to the development of a *cancer-miRNA *network that provides a global perspective on which miRNAs are dysregulated in which cancer type as well as the pattern of dysregulation, namely, upregulation or downregulation. Beside this, the network (Additional file 1) also provides miRNA's chromosomal location with start and end points, technique used to measure the expression level, fold change, P-value, cancerous samples and cell lines used in the experiment, non-cancerous samples and cell types, article references and pubmed ids. The *cancer-miRNA *network throws up several useful information such as, showing several cancer types and its associated miRNAs, identifying the miRNAs that act as hubs or those that are cancer specific. A number of hub miRNAs are also identified from the network that have a strong oncogenic or tumor suppressor characteristics, and so on. Analysis of cancer associated miRNAs based on the proposed network architecture revealed the fact that miRNAs dysregulation patterns are highly tissue dependent. A number of cancer types have been identified where a significant number of miRNAs are either upregulated or downregulated (see Table 1). The *cancer-miRNA *modules obtained from the *cancer-miRNA *network have been identified based on a computational technique of mining associations between miRNAs and cancer types. The miRNAs in a *cancer-miRNA *module depicts the fact that these miRNAs might have a chance to be combinatorially dysregulated in selected cancer tissues or cell lines. An interesting observation is that the neighboring miRNAs in a *cancer-miRNA *module may have similar dysregulation patterns in the associated cancer types. This observation is validated by reporting 12 such examples extracted from the test data set (see Table 3).

Limited work has been done towards revealing the fact that a number of miRNAs can control commonly altered regulatory pathways. However, this becomes immediately evident by accompanying the analysis of cancer-miRNA relationships in the proposed bipartite graph based network model. The importance of this network is multidimensional and raises many unaddressed questions, for example, miR-155 and miR-24-2 may be implicated in prostate cancer though such a relationship is not yet experimentally revealed. Determination of the combinatorial effects of dysregulated miRNAs in specific cancer type is still not uncovered due to the lack of knowledge on existing *cancer-miRNA *modules and it's associated common target onco/tumor suppressor genes. In this network model several cancer types have been identified where almost all the miRNAs are either upregulated or downregulated. This might be considered as an interesting research area for the researchers to discover the exact biology behind this interesting event. These observations are expected to have an intense implication in cancer and may be useful for further research.

## Methods

### Rule extraction

The association rule mining (ARM) algorithm Apriori [74] is used in order to extract the potential rules from the *cancer-miRNA *network. The ARM algorithm is decomposed into two subtasks namely (1) frequent itemset (miRNAs) generation and (2) rule generation. In the ARM: Itemset: Set of miRNAs (for example, *A, X, Y, Z*).

Support of an Itemset: Number of transactions that contains the itemset.

Frequent itemset: Itemset with support ≥ a threshold.

Confidence of a rule *X *→ *Y *provides an estimate of the conditional probability of *Y *given *X*. The probability is taken as observed frequency. The formal definitions of confidence and support are the following(1)

Find all association rules *R *in the *cancer-miRNA *network such that *Supp*(*R*) ≥ *S *(a minimal support threshold) and *Conf*(*R*) ≥ *C *(a minimal confidence threshold).

In Apriori, finds out all the frequent itemsets that are above the support threshold. The property of the Apriori algorithm is that, every subset of a frequent itemset is frequent and every superset of an infrequent itemset is infrequent. In Apriori, finds frequent itemsets of length *k *from frequent itemsets of length *k *- 1. If itemset *I *of length *k *- 1 is not frequent, then itemset *I' *of length *k *cannot be frequent, if *I *is a subset of *I'*. Hence this branch can be pruned. The steps of the first task, that is, frequent itemset generation are:

1. *C*_1 _= set of all 1-large frequent itemsets (>*S*)

2. *k *= 1

3. Find frequent itemset, *L_k_*, from *C_k_*, the set of all candidate itemsets

4. Form *C*_*k*+1 _from *L_k_*

5. *k *= *k *+ 1

6. Repeat 3 to 5 until *C_k _*is empty

7. Return ∪ *L_k_*

In rule generation, initially all the high confidence rules that have only one item in the rule consequent are extracted. These rules are then used to generate new candidate rules. For example, if {*XYZ*} → {*A*} and {*AXY*} → {*Z*} are high confidence rules, then the candidate rule {*XY*} → {*AZ*} is generated by merging the consequent of both the rules.

### Statistical analysis

To show the cancer tissue specific dysregulation patterns of miRNAs we used Fisher's exact test performed in the statistical platform R. The two dimensional contingency matrix consists of the experimental data set and the background data set. From the network, a number of upregulated and downregulated miRNAs obtained from the specific cancer tissue type are considered as the experimental data set. Rest of all the up and downregulated miRNAs from all the cancer types (except the one used in experimental data set) are considered as background data set.

A randomization test is performed for all the hub miRNAs by edge swapping procedure between cancer and miRNA such that the degree of each cancer type is preserved. Randomization test is performed 1,000 times and reject the null hypothesis that the randomized data is greater than the observed value at the 5% significance level. Similarly, for modules, each module is considered separately and the *P *value is measured by performing a randomization test. During randomization again the degree of each cancer type is preserved.

## Network visualization

The *cancer-miRNA *network is visualized by publicly available tool Pajek

http://vlado.fmf.uni-lj.si/pub/networks/pajek/

## Competing interests

The authors declare that they have no competing interests.

## Authors' contributions

SB and RM performed all analysis and wrote the manuscript. UM and MQZ provided critical insights into the article. All authors read and approved the final manuscript.

## Supplementary Material

Additional file 1 **Table S1 - Cancer-miRNA Network**. A complete list of all the miRNAs involved in different cancer types is provided in Table S1. The table contains the information of cancer type, corresponding miRNA, miRNA's chromosomal location with start and end points, miRNA's expression pattern, technique used to measure the expression level, fold change, P value, cancerous samples and cell lines used in the experiment, non-cancerous samples and cell types, article references and pubmed ids. Click here for file

Additional file 2 **Table S2 - Cancer specific miRNAs**. A list of cancer specific miRNAs, specific tissue type and dysregulation pattern are given in Table S2. Here we consider one miRNA as tissue specific (or cancer specific) if it is significantly dysregulated in at most two cancer tissue types. Click here for file
